# Differential Responses of Pediatric and Adult Primary Epithelial Cells to Human Metapneumovirus and Respiratory Syncytial Virus Infection

**DOI:** 10.3390/v17030380

**Published:** 2025-03-06

**Authors:** Pius I. Babawale, Antonieta Guerrero-Plata

**Affiliations:** Department of Pathobiological Sciences, School of Veterinary Medicine, Louisiana State University, Baton Rouge, LA 70803, USA; pbabaw1@lsu.edu

**Keywords:** HMPV, RSV, normal human bronchial epithelial (NHBE) cells, air–liquid interface culture system (ALI), human monocyte-derived dendritic cells, mo-DC, IFNs

## Abstract

Human metapneumovirus (HMPV) and respiratory syncytial virus (RSV) are pneumoviruses causing lower respiratory tract infections, primarily in infants and children rather than in healthy adults. Human bronchial epithelial cells serve as a viral replication target and source of the innate immune response to these viruses. To better understand the immune responses induced by RSV and HMPV in the pediatric airway epithelium, we comparatively studied pediatric and adult epithelial responses. We used normal human bronchial epithelial (NHBE) cells cultured in an air–liquid interface culture system (ALI), which helps to mimic the architecture of the human lower respiratory tract epithelium. Our results demonstrate differential viral replication patterns and reduced interferons; and inflammatory cytokines’ expression in pediatric cells compared to adult cells. However, pediatric epithelial cells expressed an increased mucus response and induced a stronger pro-inflammatory response in monocyte-derived dendritic cells. These findings reveal age-dependent immune epithelial responses that may contribute to more severe infections by HMPV and RSV.

## 1. Introduction

Human metapneumovirus (HMPV) and human respiratory syncytial virus (RSV, HRSV) are respiratory viruses with negative-sense single-stranded RNA genomes belonging to the *Pneumoviridae* family [1,2]. These two viruses share the same seasonal distribution, present with similar symptoms, and affect the same target populations: children, the elderly, and immunocompromised individuals [3]. The replication of these two viruses starts in the upper respiratory tract. It advances to the lower respiratory epithelium, where the pattern recognition receptors can sense the viral molecular pattern and induce the transcription of antiviral and inflammatory cytokines. In children, RSV and HMPV are a common cause of acute respiratory tract infections and lower respiratory tract infections (LRTI), like bronchiolitis and pneumonia, leading to hospitalization [4,5,6]. There are currently no specific antiviral treatments or vaccines for HMPV. Still, recently, there have been advances in RSV vaccine development, which have led to the approval of RSV vaccines to be used for the elderly (those 60 years old or older) and for pregnant women administered between week 32 and week 36 of their pregnancy to prevent RSV in babies from birth through 6 months of age. However, there is still no RSV vaccine for the pediatric population [3]. The unsuccessful RSV vaccine trial in the 1960s evidenced the challenges of generating pediatric vaccines for RSV and warranted further research analyzing the immune response in younger individuals.

Epithelial cells are the main target for RSV and HMPV replication [7,8,9,10] and are activated by these viral infections [11]. The respiratory epithelium involves the production of mucus and the induction of interferons (IFNs) and pro-inflammatory cytokines, which can activate immune cells, including dendritic cells (DCs), aiding in lung defense [12]. However, the response of primary human respiratory epithelial cells to RSV and HMPV infections is understudied since most of the in vitro responses of epithelial cells to HMPV and RSV have been studied in vitro using cell lines.

In this work, we compared the response of pediatric and adult primary normal bronchial epithelial (NHBE) cells to RSV and HMPV grown in an air–liquid interface (ALI) culture system. We demonstrated a differential response when analyzing multiple parameters, including the susceptibility of epithelial cells to RSV and HMPV infection, the expression of IFNs, IFN-stimulated genes (ISGs), cytokines, and mucins. Furthermore, we also determined the contribution of epithelial cells to activating monocyte-derived dendritic cells (mo-DC) in the context of differential age responses. These findings highlight the importance of elucidating the host response differences between children and adults infected by these pneumoviruses.

## 2. Materials and Methods

### 2.1. Cell Culture

Normal human bronchial epithelial cells (NHBE) were purchased from Lonza Bioscience (Walkersville, MD, USA). Cells included a pediatric age group (17 months to 7 years old) and an adult age group (32 to 48 years old). The cells were seeded on a 0.4 μm pore size Transwell inserted into a 24-well plate containing Pneumacult Ex Plus Basal Medium (STEMCELL Technologies, Cambridge, MA, USA) at 37 °C under a 5% CO_2_ atmosphere. After the cells were fully confluent, they were airlifted into an air–liquid interface (ALI) culture system. The basal medium was replaced with Pneumacult ALI complete medium (STEMCELL Technologies, Cambridge, MA, USA) and allowed to differentiate for 4 weeks. The ALI medium was changed every other day throughout the 4 weeks of differentiation. After 4 weeks, a fully differentiated pseudostratified columnar epithelial tissue was established.

HEp-2 and LLC-MK2 cell lines (ATCC CCL23, CCL-7, Manassas, VA, USA) were cultured in MEM/EBSS medium (Hyclone, Logan, UT, USA) supplemented with 10% FBS (Gibco, Gaithersburg, MD, USA) and 1% penicillin-streptomycin (Gibco, Gaithersburg, MD, USA). These cells were used to propagate and titrate the viral pools, as indicated below.

### 2.2. Establishment of Monocyte-Derived Dendritic Cells

Monocyte-derived dendritic cells (mo-DCs) were generated from human peripheral blood mononuclear cells (PBMCs) from healthy donors (Our Lady of the Lake Blood Donor Center, Baton Rouge, LA, USA) as previously reported [13]. Briefly, adherent PBMCs were cultured in an RPMI-supplemented medium containing GM-CSF (100 ng/mL) (Peprotech, Cranbury, NJ, USA) and IL-4 (20 ng/mL) (R&D Systems, Minneapolis, MN, USA) and allowed to differentiate for 7 days.

### 2.3. Virus Stock

HMPV strain CAN 97-83 was obtained from the Centers for Disease Control (CDC), Atlanta, GA, USA, and was propagated in LLC-MK2 cells in MEM containing 1 μg trypsin/mL (Worthington Biochemicals, Lakewood, NJ, USA). HMPV was purified by polyethylene glycol precipitation, followed by centrifugation on a 60% sucrose cushion [14,15]. HMPV viral pools were titrated by a combined method of methylcellulose plaque assay and cell-based immunoassay in LLC-MK2 cells, as previously reported [13]. RSV strain A2 was purchased from the American Type Culture Collection ((ATCC), Manassas, VA, USA) and propagated in HEp2 cells. RSV was purified by polyethylene glycol precipitation, followed by centrifugation on 35–65% discontinuous sucrose gradients [16]. RSV viral pools were titrated by methylcellulose plaque assay in HEp2 cells [17].

### 2.4. Viral Infection

Fully differentiated NHBE cells were washed twice with 150 μL of Dulbecco’s phosphate buffered saline (DPBS) and then infected with either HMPV or RSV at a multiplicity of infection (MOI) of 0.02 on the apical side. After 2 h of adsorption at 37 °C, the inoculum was removed, and the culture continued to maintain an ALI state. Cells were lysed at different time points after infection, and lysates were collected for further analysis.

### 2.5. RNA Extraction

Cells were lysed at different time points, and RNA was extracted with an RNeasy-plus kit (Qiagen, Germantown, MD, USA), according to the manufacturer’s recommendations.

### 2.6. Quantitative Real-Time Reverse Transcription PCR (qRT-PCR)

The expressions of IFN, pro-inflammatory cytokines, and mucins were quantified by qRT-PCR. cDNA was synthesized from total RNA using the LunaScript RT SuperMix Kit (New England Biolabs, Ipswich, MA, USA) according to the manufacturer’s instructions. cDNA fragments of interest were amplified using the PowerTrack SYBR Green Master Mix (ThermoFisher Scientific, Waltham, MA, USA). Primers for IFN-α2, IFN-β, IFN-ε, IFN-ω, IFN-λ1, IFN-λ2/3, interleukin-1β (IL-1β), IL-6, IL-8, tumor necrosis factor-α (TNF-α), IFN-induced protein tetratricopeptide repeats 1 (IFIT1), IFIT2, IFIT3, Myxovirus resistance protein 1 (MX1), IFN-stimulated gene 15 (ISG15), 2′-5′-oligoadenylate synthetase 1 (OAS1), IL-25, IL-33, thymic stromal lymphopoietin (TSLP), and mucins (MUC)5AC, MUC5B, and GAPDH (from Integrated DNA Technologies (IDT, Newark, NJ, USA)) were run on the QuantStudio™ 12k PCR system (Applied Biosystems, Foster City, CA, USA). The CT method (ΔΔCT) was used to quantitate the expression of target genes, which were normalized to the endogenous reference (GAPDH) expression levels of transcripts from corresponding uninfected or infected cells. The specificity of the reaction was determined by melting curve analysis of the amplification products.

### 2.7. Multiplex Cytokine Expression Analysis

Cytokine release was determined from apical washes of the ALI cultures with DPBS, and the samples were stored at −80 °C until use. Cytokine levels were evaluated using the LEGENDplex^TM^ (13-plex) Human Inflammation Panel 1 (BioLegend San Diego, CA, USA) according to the manufacturer’s instructions. Samples were acquired using an LSR Fortessa flow cytometer (BD Biosciences San Diego, CA, USA). The data analysis was conducted and cytokine concentrations were determined using the cloud-based LEGENDplex^TM^ Data Analysis Software Suite.

### 2.8. Statistical Analysis

Statistical analyses were calculated by two-way analysis of variance (ANOVA) or Kruskal–Wallis tests followed by post-hoc tests to ascertain the differences between the tested conditions using GraphPad Prism version 10.4.1 (GraphPad Software, Boston, MA, USA).

## 3. Results

### 3.1. Differential Susceptibility of Pediatric and Adult NHBE Cells to HMPV and RSV Infection

**Figure 1:**
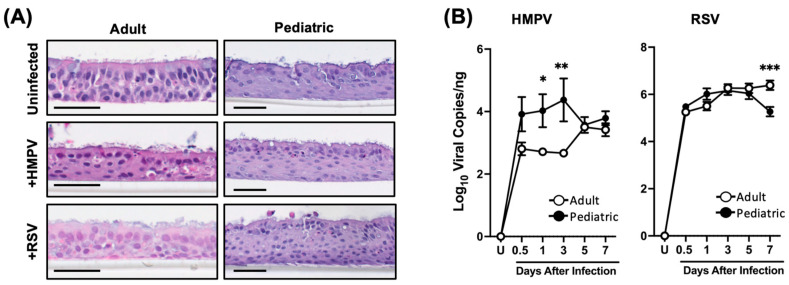
Age-related differences in NHBE cell susceptibility to HMPV and RSV infection. NHBE cells from pediatric and adult donors cultured at the air–liquid interface (ALI) and infected with RSV or HMPV. (**A**) After 7 days of HMPV and RSV infection, cells were stained with H&E staining to assess cell morphology; Scale bar = 50 μm. (**B**) Kinetics of viral copy numbers. Data represent mean ± SEM from three donors for each age group. Statistical significance was determined using two-way ANOVA with Šídák’s multiple comparisons test (* *p* < 0.05, ** *p* < 0.01, *** *p* < 0.001).

In order to investigate the response of human airway epithelial cells from children and adults to RSV and HMPV, NHBE cells from pediatric and adult donors were fully differentiated into pseudostratified columnar epithelial tissue for 4 weeks in an ALI culture. The cells were then infected with HMPV or RSV at an MOI of 0.02 via the apical side since the infection and replication of these viruses primarily occur in the ciliated cells of the airway epithelium [18,19,20]. To analyze the morphology of the infected cultures, cells were collected on day 7 after infection. The samples were paraffinized, sectioned, and stained with hematoxylin and eosin (H&E). As shown in Figure 1A, the morphologies of uninfected cells from both age groups were similar. The cells were fully differentiated, showing ciliated cells on the apical side. When comparing virus-infected cells from both groups of individuals, we observed slightly more damage in those infected with RSV than with HMPV since the ciliated cells were less evident.

Regarding the viral susceptibility of the cells from the two age groups, samples from the infected cells were collected at different time points. As shown in Figure 1B, we observed that while HMPV replicates in both groups of cells, there was a significantly greater number of viral copies from pediatric cells than from adult ones. The viral gene copy number in adults at 0.5 days was 2.8 Log10_10_ copies/ng of RNA, and similar values were maintained up to day 3 (2.66 Log_10_ viral copies/ng), followed by a peak on day 5 with 3.5 Log_10_ copies/ng. On the other hand, the HMPV viral gene copy number in pediatric cells constantly increased from 3.91 Log_10_ viral copies/ng on day 0.5, peaking on day 3 (4.37 Log_10_ viral copies/ng) and decreasing to 3.57 Log_10_ copies on day 5, resulting in a replication difference of 1.71 Log_10_ HMPV viral copies in pediatric cells vs adult cells on day 3. For RSV infection, the viral gene expression between the two age groups did not change significantly up to day 5 after infection, when the viral copy numbers were about 6.1 Log_10_ copies/ng, after which the copy numbers in the pediatric cells declined to 5.2 Log_10_ copies/ng and increased in the adult cells to 6.3 Log_10_ copies/ng. Overall, these data indicate no major morphological differences between epithelial cells from the two age groups. Moreover, HMPV replicated more in pediatric epithelial cells than in adult cells, while RSV has similar replication kinetics in epithelial cells in both age groups.

### 3.2. Pediatric NHBE Cells Express IFN Responses That Are Distinct from Those in Adult Cells upon Infection with RSV or HMPV

**Figure 2:**
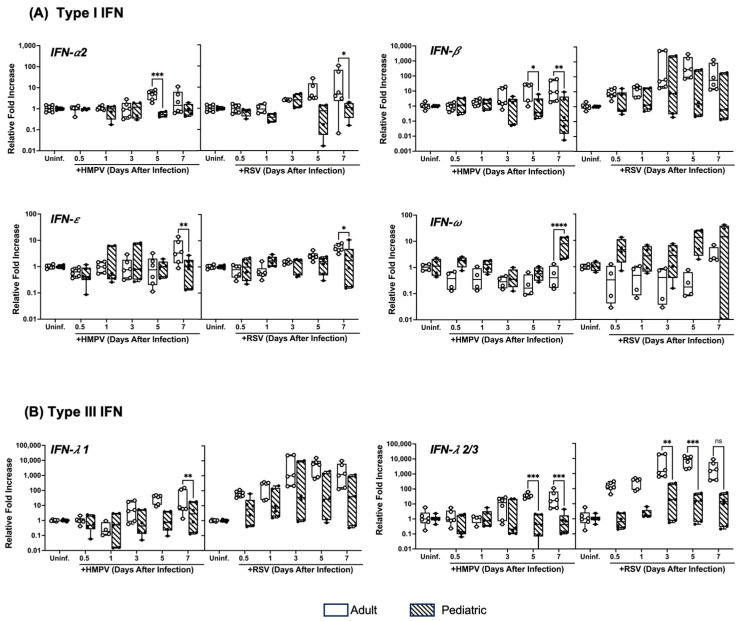
IFN responses of pediatric and adult NHBE cells to RSV and HMPV infection. NHBE cells were infected with RSV or HMPV. Gene expression of (**A**) type I IFNs (IFN-α2, IFN-β, IFN-ε, and IFN-ω) and (**B**) type III IFNs (IFN-λ1 and IFN-λ2/3) was assessed by RT-qPCR at different time points. Statistical significance was determined using two-way ANOVA with Šídák’s multiple comparisons test (* *p* < 0.05, ** *p* < 0.01, *** *p* < 0.001, **** *p* < 0.0001). Non-significant (ns).

Based on the differences observed in the viral replication kinetics between the two age groups, we analyzed the antiviral response induced by HMPV or RSV infection in pediatric and adult epithelial cells by characterizing the IFN responses. We determined IFN-α2, IFN-β, IFN-ε, and IFN-ω were part of the type I IFN response. For type III IFNs, we assessed the induction of IFN-λ1 and IFN-λ2/3. NHBE cells were infected with RSV or HMPV, and the expression of the IFN response was evaluated at the same time points as in Figure 1B. As shown in Figure 2A, RSV and HMPV induced a stronger IFN-α2 response in adults than in children between days 5 and 7 after the infection of the cells. A similar effect was observed with the expression of IFN-ε, in which a higher expression was observed on day 7 in adult cells than in the pediatric epithelium. The response of IFN-β also exhibited differences between the age groups, mainly for HMPV, in which the adult cells responded with higher expression of IFN-β on days 5 and 7 than pediatric cells. However, no significant differences were observed in the expression of IFN-β when infected with RSV. Interestingly, the expression of IFN-ω induced by RSV and HMPV was higher in the pediatric cells than in the cells from adults. When analyzing the response of type III IFNs, we observed that the adult cells express more IFN-λ1 and IFN-λ2/3 than the pediatric cells after RSV or HMPV infection (Figure 2B). Overall, we observed higher type I and III IFN induction by HMPV and RSV in adult cells than in pediatric cells, except for IFN-ω, which was more induced in the pediatric cells.

### 3.3. Differential Expression of ISGs in NHBE Cells from Adults and Children Infected with HMPV and RSV

**Figure 3:**
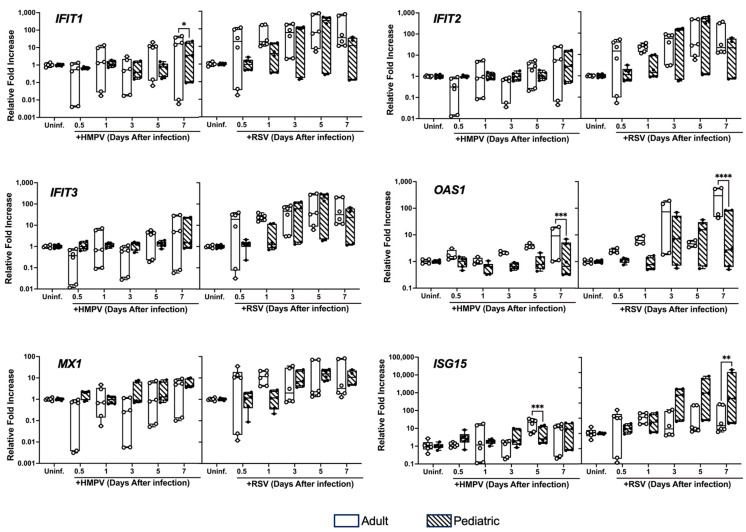
Differential expression of ISGs by pediatric and adult NHBE cells. Adult and pediatric NHBE cells were differentiated in ALI culture and infected with RSV and HMPV. RNA samples were collected at different time points and analyzed for expression of key ISGs (IFIT1, IFIT2, IFIT3, OAS1, MX1, and ISG15) by RT-qPCR. Statistical significance was determined using two-way ANOVA with Šídák’s multiple comparisons test (* *p* < 0.05, ** *p* < 0.01, *** *p* < 0.001, **** *p* < 0.0001).

Next, we analyzed the induction of the antiviral IFN-stimulated genes (ISGs), including IFIT1, IFIT2, IFIT3, OAS1, MX1, and ISG15. NHBE cells were infected as described above, and the expression of the ISGs was assessed by RT-qPCR. As shown in Figure 3, our results demonstrated no significant difference in the induction of IFIT2, IFIT3, and MX1 between the two age groups upon HMPV and RSV infection. Nevertheless, the expression level was higher for both age groups in the RSV-infected cells than in the HMPV-infected cells. Regarding the expression of IFIT1, we observed a significant increase in the adult epithelial cells on day 7 of HMPV infection compared to the pediatric cells, but no difference for the RSV-infected cells. The expression of OAS1 was more induced by both viruses in the adult cells than in the pediatric cells on day 7. Similarly, the induction of ISG15 by HMPV was more induced in adult cells on day 5 after infection. Interestingly, we observed a significant increase in ISG15 in the RSV-infected pediatric group compared to the adult cells on day 7. These results suggest that HMPV and RSV induced a differential antiviral response in the pediatric and adult epithelium.

### 3.4. Differential Cytokine Expression of NHBE Cells from Adults and Children Infected with HMPV and RSV

**Figure 4:**
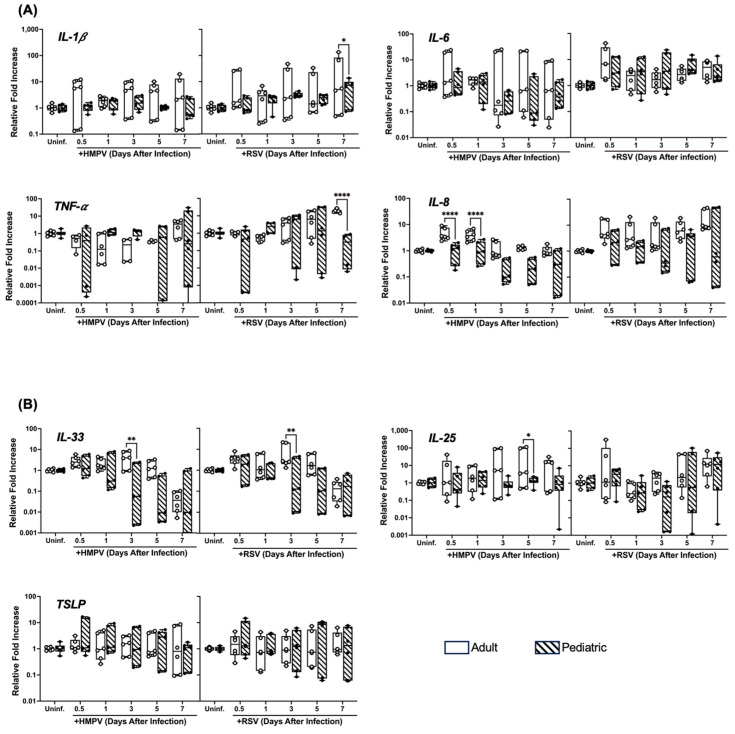
Cytokine responses in NHBE cells infected with HMPV or RSV. Adult and pediatric NHBE cells were differentiated in ALI culture and infected with RSV or HMPV. RNA samples were collected at different time points and analyzed by RT-qPCR for the expression of (**A**) pro-inflammatory cytokines and (**B**) epithelial alarmins. Statistical significance was determined using two-way ANOVA with Šídák’s multiple comparisons test (* *p* < 0.05, ** *p* < 0.01, **** *p* < 0.0001).

Airway epithelial cells produce a variety of cytokines upon viral infections [21,22], representing a central role in the inflammatory process against HMPV and RSV [23,24]. Therefore, we measured the induction of pro-inflammatory cytokines IL-1β, IL-6, IL-8, and TNF-α (Figure 4A). NHBE cells from pediatric and adult donors were infected with HMPV or RSV. The expression of cytokines was determined by RT-qPCR at different time points after infection. Our results indicate that the expression of IL-1β was induced more in the adult cells than in the pediatric cells by both viruses, observing a significant difference in RSV on day 7. A similar effect was observed on the expression of IL-8, in which a greater response was elicited by the cells from the adults, which was significant 12 and 24 h after the cells were infected with HMPV. IL-6 expression was induced more in the adult cells when infected with HMPV, but not much difference was observed between both age groups after RSV infection. Regarding the expression of TNF-α, no significant difference was shown, except in cells after 7 days after RSV infection.

Furthermore, since HMPV and RSV infection in children has been reported to drive type 2 immune responses, we analyzed the response of epithelial alarmins IL-25, IL-33, and TSLP, which play significant roles in the pathophysiology of asthma by promoting inflammation and airway hyperresponsiveness [25,26,27]. As shown in Figure 4B, RSV and HMPV induced significantly more IL-33 in the adult cells on day 3 after infection. However, only HMPV induced significant differences in IL-25 on day 5 between the two age groups. No major differences were observed in the TSLP expression between the two age groups. However, a trend of a higher expression of TSLP in the cells from children by RSV and HMPV infection was observed at 12 h.

**Figure 5:**
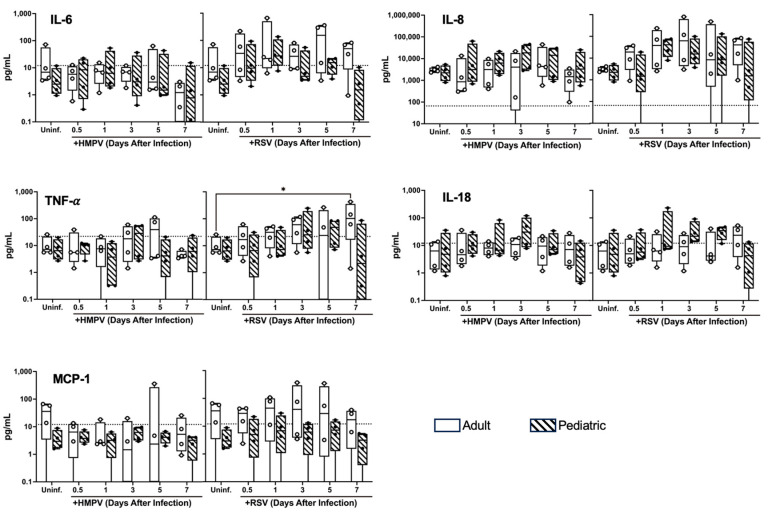
Cytokine release from human epithelial cells infected with HMPV or RSV. Pediatric and adult NHBE cells were grown in ALI culture and infected with HMPV or RSV. Apical washes were collected at different time points after infection, and concentration of cytokines was determined by LEGENDplex multiplex immunoassay. Statistical significance was determined using two-way ANOVA with Dunnett’s multiple comparisons test (* *p* < 0.05).

In addition, we analyzed the inflammatory cytokine release between the age groups. The expression of IL-1β, IFN-α2, IFN-γ, TNF-α, MCP-1 (CCL2), IL-6, IL-8 (CXCL8), IL-10, IL-12p70, IL-17A, IL-18, IL-23, and IL-33 was assessed in apical washes at different time points after infection using a 13-plex immunoassay. Our results in Figure 5 show that both viruses induced the expression of IL-6, IL-8, TNF-α, IL-18, and MCP-1 with a preponderance of IL-8 production. Also, we found that RSV induced higher levels of cytokines than HMPV. Moreover, we observed that, except for IL-18, the adult cells had a trend of expressing higher levels of IL-6, IL-8, TNF-α, and MCP-1 than the pediatric cells, with a significant increase in TNF-α on day 7 after RSV infection. The expressions of IL-1β, IFN-α2, IFN-γ, IL-12p70, IL-17A, IL-10, IL-33, and IL-23 were under the lower limit of detection. Together, these data indicate that adult and pediatric bronchial epithelial cells have a differential expression of cytokines in response to pneumovirus infection.

### 3.5. Higher Mucin Expression in RSV-Infected Pediatric NHBE Cells than in Adult Cells

**Figure 6:**
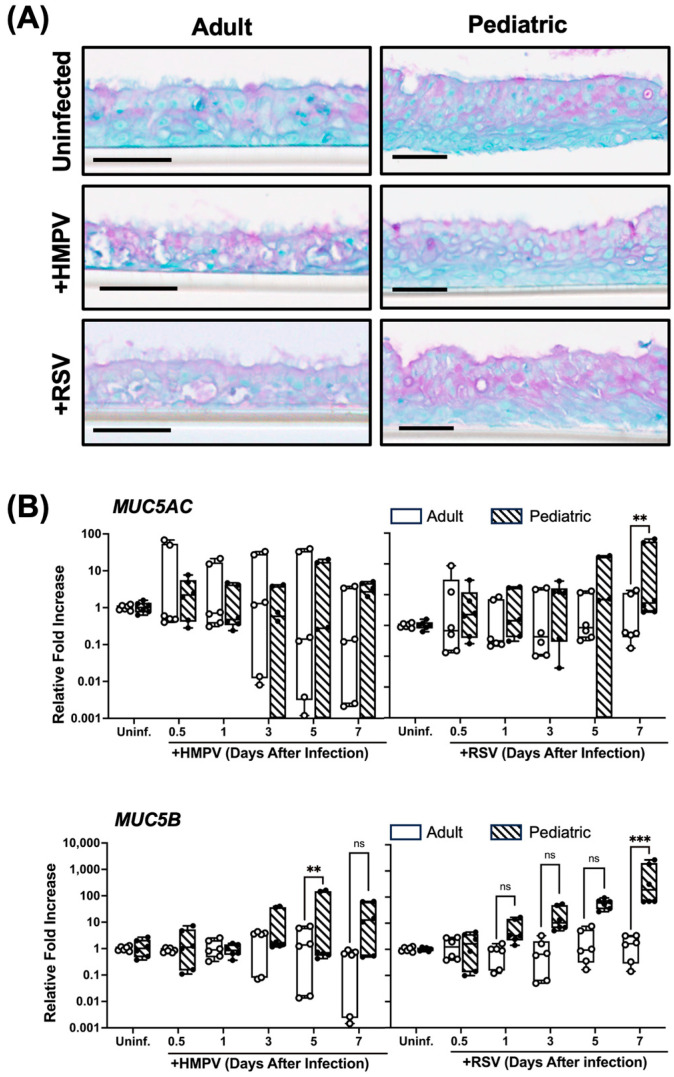
Mucin expression induced by RSV and HMPV in pediatric and adult NHBE cells. NHBE cells were differentiated in ALI culture and infected with RSV and HMPV. (**A**) Cells were stained with PAS histological staining on day 7 after infection. Scale bar = 50 μm. (**B**) Further analysis by RT-qPCR assessed the expression of *MUC5AC* and *MUC5B* levels. Statistical significance was determined using two-way ANOVA with Šídák’s multiple comparisons test (** *p* < 0.01, *** *p* < 0.001. Non-significant (ns).

Mucus and mucins are critical components of the airway epithelium defense system, as they provide a physical barrier that traps and facilitates the clearance of pathogens [28,29]. However, mucin overexpression has been linked with increased disease severity in RSV-infected children [30], and mucus has been found in children infected with HMPV [31]. Therefore, the mucus response in NHBE cells was investigated. Cells from children and adults were infected and processed for periodic acid–Schiff (PAS) staining and MUC5AC and MUC5B expression. At the basal level in uninfected cells, no evident difference in PAS staining was found between the adult and pediatric tissue (Figure 6A). A similar effect was observed when cells were infected with HMPV, in which no difference was shown in the staining between the two age groups. However, when cells were infected with RSV, we observed that the pediatric tissue showed greater PAS staining than the adult tissue. Furthermore, the assessment of the expressions of *MUC5AC* and *MUC5B*, two secreted mucins in the respiratory epithelium, indicated that RSV induced higher transcript levels of mucins in the pediatric cells than in the adult ones, particularly on day 7 after RSV infection (Figure 6B). When the cells were infected with HMPV, MUC5B was more induced in pediatric cells compared to adult cells on day 5 after infection, but no significant changes were observed in the expression of MUC5AC (Figure 6B). Overall, these data suggest that RSV induces more mucus in cells from children than from adults.

### 3.6. Infection of Pediatric NHBE Cells by HMPV or RSV Drives Higher Stimulation of Inflammatory Cytokines in Dendritic Cells

**Figure 7:**
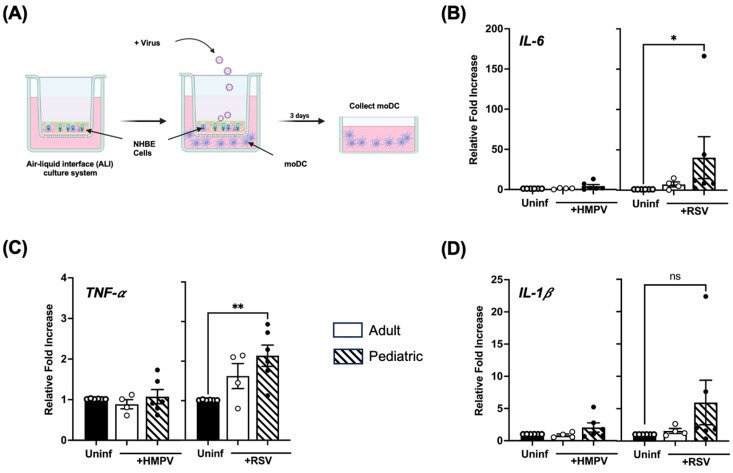
Induction of inflammatory cytokines in mo-DCs co-cultured with either pediatric or adult NHBE cells. mo-DCs were co-cultured with NHBE cells from either pediatric or adult donors infected with HMPV or RSV. (**A**) Schematic representation of the co-culture setup: Fully differentiated NHBE cells were infected with HMPV or RSV and cultured with mo-DCs for 3 days. mo-DCs were analyzed for the expression of (**B**) IL-6, (**C**) TNF-α, and (**D**) IL-1β by RT-qPCR. Data represent mean ± SEM, *n* = 4–6. Statistical significance was determined using the Kruskal–Wallis test (* *p* < 0.05, ** *p* < 0.01); non-significant (ns).

The human lung environment includes a variety of immune cells, which, together with infected epithelial cells, can induce an immune response against respiratory viruses. Dendritic cells, as one of these immune cells, are the primary antigen-presenting cells, and they play a crucial role in mediating immune responses between innate and adaptive immunity. Signals from infected respiratory epithelial cells, such as TSLP, which we saw in our previous results on its transient upregulation in pediatric cells (Figure 4B), can influence the immune responses of DCs, especially their cytokine profiles [32,33,34]. Therefore, we co-cultured virus-infected NHBE cells from both age groups with human mo-DC, as illustrated in Figure 7A. We assessed the pro-inflammatory cytokine profile expressed by the mo-DC stimulated by infected NHBE cells, measuring the induction of IL-1β, IL-6, and TNF-α by RT-qPCR. Our data showed that IL-6 gene expression significantly increased in mo-DC co-cultured with RSV-infected NHBE cells from children but not from adult cells. Compared with mo-DC exposed to uninfected NHBE cells, we observed that RSV-infected cells from children induced IL-6 ~40 times in mo-DCs (Figure 7B). Moreover, compared to uninfected cells, RSV-infected pediatric cells induced significant expression of TNF-α in mo-DCs (~a two-fold increase) (Figure 7C). The expression of IL-1β was more induced by pediatric cells infected with RSV, but no statistical difference was found (Figure 7D). No significant IL-6, TNF-α, and IL-1β induction was observed in mo-DCs when co-cultured with HMPV-infected epithelial cells. Overall, our data suggest that pediatric-infected epithelial cells stimulate an inflammatory response in DCs more than in adult cells, particularly in the case of RSV-infected cells.

## 4. Discussion

HMPV and RSV are two pneumoviruses that affect the same target populations: pediatric populations, the elderly, and immunocompromised adults [3,35]. However, they also affect less healthy adults. There are efforts to understand why children develop more severe RSV and HMPV infections compared to adults, which is in contrast to some other respiratory viral infections, like COVID-19, by which children are less affected [36,37,38]. Nevertheless, the pediatric immune response induced by RSV and HMPV infection remains largely unknown. In this paper, we compare the susceptibility and immune response of the pediatric and adult airway epithelium to either HMPV or RSV infection, demonstrating age-dependent immune differences in the interaction of these viruses with respiratory epithelial cells. To the best of our knowledge, this is the first report to define the differential activation of pediatric and adult human epithelial cells by RSV and HMPV infection.

Respiratory epithelial cells are the main target of HMPV and RSV replication [10,39]. In this study, we used primary adult and pediatric human bronchial epithelial cells in an ALI culture, which mimics the human airway epithelium, and assessed their viral susceptibility. Our findings revealed the distinct replication kinetics of HMPV and RSV in pediatric and adult NHBE cells while maintaining morphological consistency across both age groups. Importantly, we observed a higher HMPV viral load in pediatric cells compared to adult cells. That is relevant since a high HMPV viral load is associated with more severe disease in infants and young children [40,41,42]. These findings suggest that age-specific host cell responses play a critical role in shaping viral replication dynamics.

The antiviral response, including IFN production, is one of the host cell responses to control viral infections. Our findings indicate that adult NHBE cells consistently exhibited stronger responses of IFN-α2, IFN-β, IFN-ε, IFN-λ1, and IFN-λ2/3 compared to pediatric cells. This is in line with the reduced response of IFN-α and IFN-β induced by RSV infection in the neonatal mouse model [43,44]. On the other hand, IFN-ω expression was more induced in pediatric cells than in adult cells. This unique pattern suggests that IFN-ω may compensate for pediatric antiviral responses to HMPV and RSV. Nevertheless, the antiviral effect of IFN-ω on human respiratory viruses is not well established. Still, its antiviral effect has been reported in avian influenza infection [45], suggesting a possible antiviral effect against other respiratory viruses. Thus, future research investigating the susceptibility of HMPV and RSV to IFN-ω is warranted. ISGs are induced by IFNs, are the effector molecules in the antiviral response, and are crucial downstream mediators of type I and type III IFN signaling [46,47]. After the IFNs are released, they bind their corresponding receptors to the same cell or bystander cells. These events initiate signaling pathways that stimulate the production of ISGs that trigger an antiviral state to suppress viral replication. Here, we observed that several ISGs, including IFIT1, IFIT2, IFIT3, and MX1, exhibited limited differential expression between the two age groups in response to both viruses. These findings suggest that these ISGs are uniformly regulated across age groups during HMPV and RSV infection. However, adult cells showed significantly higher OAS1 expression on day 7 than pediatric cells for both viruses, suggesting a more robust antiviral mechanism in adults. Notably, pediatric cells exhibited a significant increase in ISG15 expression on day 7 of RSV infection. This observation may reflect an age-specific immune response or compensatory mechanism to address the overall weaker antiviral signaling in pediatric epithelial cells. ISG15 is a potent antiviral protein capable of significantly inhibiting the replication of a wide range of viruses, including influenza A virus, human immunodeficiency virus-1 (HIV-1), and herpes simplex virus, through direct interference with viral processes within host cells [48,49,50]. However, its antiviral efficacy is virus-specific and influenced by the host immune response, with certain viruses demonstrating limited susceptibility to ISG15-mediated modulation. Collectively, our findings indicate that the IFN and antiviral responses induced in epithelial cells are age-dependent and differentially induced by RSV and HMPV. Nevertheless, their correlation with viral load in the infected cells is unclear. We acknowledge that other factors not contained in this study could contribute to the greater HMPV viral replication observed in the pediatric cells. For instance, pediatric cells may have a higher expression of cell surface receptors or co-factors that facilitate HMPV entry and increase infectivity. Therefore, future research is warranted to understand the differential HMPV viral load between adults and infants.

Pro-inflammatory cytokines can modulate the recruitment and activation of immune cells to the site of infection, which can influence viral clearance and the severity of the disease [51,52]. The capacity of epithelial cells to induce these cytokines can vary significantly with age. When we compared the expression of inflammatory cytokine response of adult and pediatric cells upon HMPV and RSV infection, we observed that adult cells produced more pro-inflammatory cytokines (IL-1β, IL-6, IL-8, and TNF-α) following infection. Similarly, data reported in mice indicate that neonatal mice are less responsive to RSV and HMPV than adult mice [53] since neonates produce fewer immune mediators than adult mice, such as those cytokines that maintain a robust type 1 immune response [54]. When analyzing protein cytokine release, we observed that RSV induced a stronger response than HMPV, which is consistent with findings from clinical samples in which infants infected with RSV had a higher expression of inflammatory cytokines than those infected with HMPV [55]. Moreover, RSV induced a stronger TNF-α expression in adult cells, suggesting that adult cells may have a stronger pro-inflammatory response to these pathogens. We also observed that IL-8 was the most highly induced inflammatory cytokine by RSV and HMPV in both age groups. This finding is in agreement with clinical data from nasal washes from infants infected with RSV or HMPV, in which IL-8 was the highest induced inflammatory cytokine compared with the expression of IL-1β, IL-6, IL-10, IL-12, and TNF-α [55]. IL-8 is a potent chemoattractant for neutrophils, guiding them to sites of infection or inflammation. During viral infections, IL-8 can contribute to excessive neutrophil infiltration, and while neutrophils are essential for antiviral immunity, their excessive activation can lead to tissue damage, bronchiolitis, and chronic lung conditions [56]. We also observed lower baseline protein levels of IL-6 in pediatric cells than in adult cells, which is in line with studies showing that IL-6 levels in children, particularly during the second year of life, are lower than those in adults [57]. Likewise, MCP-1 (CCL2), a chemokine involved in monocyte recruitment, was also higher at baseline in adult NHBE cells than in pediatric cells. These findings could indicate a delayed pro-inflammatory state in pediatrics, as the immune system is skewed away from a pro-inflammatory state during early life to prevent damage to developing tissues like the lungs [58]. IL-25, IL-33, and TSLP are epithelial-derived cytokines that serve as an initial defense mechanism against airway epithelial infection and irritation, primarily driving type 2 inflammatory responses and playing a key role in bridging innate and adaptive immunity [59,60]. These cytokines have been implicated in asthma and bronchoconstriction in children [59,61] and play a role in the pathogenesis and inflammatory response to RSV and HMPV [33,62,63]. We found an increase in the gene expression of epithelial-derived IL-25 and IL-33 on day 3 after infection in adult cells. However, no significant changes were observed in the expression of TSLP. Overall, the differences in cytokine profiles may influence the severity and progression of the inflammatory response in children versus adults during HMPV or RSV infections.

Secreted mucins, particularly MUC5AC and MUC5B, play a vital role in the innate defense mechanism of the respiratory epithelium by trapping pathogens and facilitating their clearance [64,65]. However, excessive mucus production has been implicated in the exacerbation of disease severity by contributing to airway obstruction and impaired gas exchange [66]. We observed no significant difference in basal mucin levels between uninfected pediatric and adult epithelia, as indicated by their comparable PAS staining. However, the RSV infection of pediatric tissues induced a more pronounced increase in mucin production compared to adult tissues. This was evident from both the enhanced PAS staining and the upregulation of MUC5AC and MUC5B transcripts in pediatric cells. In contrast, HMPV infection resulted in similar PAS staining levels between age groups, but a transcript analysis revealed greater MUC5B induction in pediatric cells. These findings suggest that pediatric NHBE cells exhibit a heightened mucinogenic response to RSV and HMPV compared to adult cells, potentially contributing to increased mucus plugging and disease severity in younger patients. Further research into the regulation of mucin expression and its downstream effects could help develop therapeutic strategies to mitigate airway obstruction in pediatric patients during HMPV and RSV infections.

Dendritic cells are crucial antigen-presenting cells, bridging innate and adaptive immunity [67,68,69]. Infected epithelial cells release cytokines and chemokines that signal to dendritic cells. These signals from epithelial cells can determine the inflammatory profile of dendritic cells, which in turn determines the polarization of naive CD4+ T cells [70], as their differentiation into effector T-cells with specific functions is based on molecular signals provided by dendritic cells. Our findings demonstrate that pediatric NHBE cells infected with HMPV or RSV drive a stronger inflammatory response in mo-DCs than adult NHBE cells. Notably, IL-6 and TNF-α induction was significantly more elevated in mo-DCs co-cultured with pediatric epithelial cells infected by RSV. On the other hand, no major changes were observed for IL-1β expression. These findings suggest that pediatric epithelial cells can activate DCs, predisposing them to induce a Th-2 response, as it has been shown that lung Th2 response is mediated by the DC-mediated inhibition of Th1 responses via IL-6 production [71,72]. Moreover, signals like TNF-α can enhance Th2 immune responses in asthma pathogenesis [73] and RSV-induced disease [74]. This age-related difference could contribute to the heightened inflammation and immune dysregulation observed in pediatric RSV and HMPV infections [75,76].

## 5. Conclusions

This study demonstrates an age-dependent difference in the immune responses of epithelial cells upon infection with HMPV and RSV. Pediatric NHBE cells exhibited a distinct cytokine, IFN, and mucus profile compared to adult cells, marked by lower levels of inflammatory cytokines and IFN response and higher levels of mucus production and DC activation, which may contribute to the more severe clinical manifestations in children during HMPV and RSV infections.

## Data Availability

The data supporting the conclusions of this research manuscript are all present within the article.
